# ECMO improves survival following cardiogenic shock due to carbon monoxide poisoning - an experimental porcine model

**DOI:** 10.1186/s13049-018-0570-6

**Published:** 2018-11-22

**Authors:** Carsten Simonsen, Sigridur O. Magnusdottir, Jan J. Andreasen, Marianne Cathrine Rohde, Benedict Kjærgaard

**Affiliations:** 1 Department of Cardiothoracic Surgery, Aalborg University Hospital, Hobrovej 18-22, 9000 Aalborg, Denmark; 2 Department of Clinical Medicine, Aalborg University, Sdr. Skovvej 15, 9000 Aalborg, Denmark; 3 Biomedical Research Laboratory, Aalborg University Hospital North, Ladegårdsgade 3, 9000 Aalborg, Denmark; 4 Department of Forensic Medicine, Aarhus University, Palle Juul-Jensens Boulevard 99, 8200 Aarhus, Denmark

**Keywords:** Carbon monoxide poisoning, Smoke poisoning, Extracorporeal membrane oxygenation, Hyperbaric oxygenation, Cardiac failure, Respiratory failure, Pulmonary vascular resistance

## Abstract

**Background:**

Severe intoxication with carbon monoxide (CO) is extremely lethal and causes numerous deaths due to cardiac or respiratory failure. Conventional intensive treatment may not be sufficient. The aim of this study was to investigate the treatment effect of extracorporeal veno-arterial extracorporeal membrane oxygenation (ECMO) following severe CO poisoning in an experimental porcine model.

**Methods:**

A total of twelve pigs were anaesthetized, routinely monitored and intoxicated by inhalation of CO until the beginning of cardiac failure and randomized to a treatment (ventilator using an FiO_2_ of 100% or ECMO). In the case of cardiac arrest, advanced resuscitation using standard guidelines was performed for at least 10 min. ECMO was also initiated in the ventilation group if the return of spontaneous circulation did not occur within 10 min. Lung tissue biopsies were obtained before and after CO intoxication.

**Results:**

All animals in the ECMO group survived; however, one had to be resuscitated due to cardiac arrest. A single animal survived in the ventilator group, but five animals suffered from cardiac arrest at an average of 11.8 min after initiation of treatment. Conventional resuscitation failed in these animals, but four animals were successfully resuscitated after the establishment of ECMO.

A significant decrease was noticed in PO_2_ with increasing HbCO, but there was no increase in pulmonary vascular resistance. No differences in H&E-stained lung tissue biopsies were observed.

**Conclusions:**

The use of ECMO following severe CO poisoning greatly improved survival compared with conventional resuscitation in an experimental porcine model. This study forms the basis for further research among patients.

## Introduction

Carbon monoxide (CO) is extremely treacherous; invisible and without smell or taste, this lethal gas overtakes people without warning. Negligible CO concentrations occur in the atmosphere, but large amounts of CO form during the insufficient combustion of organic material, and a primary risk of exposure is the inhalation of smoke from fires [1]. Other significant sources of CO poisoning include residential heat sources, suicide/attempts and occupational exposure [2–5].

Approximately 50,000 annual contacts with emergency departments in the US are due to CO poisoning, resulting in approximately 2700 deaths [6, 7]. Surviving patients may suffer from increased risk of developing neurological symptoms, e.g., extrapyramidal symptoms, encephalopathy and cardiac insufficiency [8–10].

The rationale behind all currently established treatments for CO poisoning is to elevate the partial pressure of oxygen in the blood, favouring the formation of HbO_2_ instead of HbCO, and to increase the oxygen content in the blood [11, 12]. In mild cases, this is achieved by administering supplementary normobaric oxygen (NBO) for inhalation. In more severe cases, hyperbaric oxygen treatment (HBO) is used [11]. HBO can further increase blood oxygen content, resulting in a decreased half-life of HbCO, but the protocol for when to offer HBO differs greatly internationally [13].

CO poisoning may have a profound effect on both respiratory and cardiac function, but although HBO increases blood oxygen tension and decreases HbCO half-life, the benefits of HBO remain highly debated [14–17]. Conventional intensive treatment methods may not be sufficient, and the aim of the present study was to investigate the treatment effect of extracorporeal veno-arterial extracorporeal membrane oxygenation (ECMO) following severe CO poisoning in an experimental porcine model. The porcine model was chosen due to the close resemblance between porcine and human anatomy/physiology [18]. We hypothesized that (ECMO) would improve survival.

## Methods

### Ethical statement

This study was carried out in accordance with Danish and European legislation regarding the use of animals for research purposes. The experiments were approved by the Danish Animal Experiments Inspectorate (J.nr. 2016-15-0201-01064). At all times, a veterinarian was present, and all participants had training in laboratory animal science prior to experimentation.

### Experimental animals and instrumentation

All experiments were carried out at the Biomedical Research Laboratory at Aalborg University Hospital, Denmark. The research animals were 12 female pigs (Danish Landrace) with an average weight of 48 kg (range 45–51 kg) and approximately 90 days old. The animals were housed in nearby boxes at the laboratory for acclimatization up to 7 days prior to the experiment. During this period, the animals had access to food/water and were attended to by laboratory staff several times each day. Premedication with Zoletil, an anaesthetic combination drug containing equal concentrations of Tiletamine and Zolazepam, was used. Anaesthesia, which was similar in both study groups, was maintained with continuous intravenous infusion of fentanyl and propofol based on weight, with minor adjustments to ensure that anaesthesia was sufficient. The animals were intubated using a 6.5-mm cuffed endotracheal tube and connected to a ventilator (Dameca DREAM, Rødovre, Denmark). Tidal volume was calculated using 8 mL/kg and the respiratory rate (RR) was adjusted according to blood CO_2_ levels (14–17/min) and reduced whenever ECMO was running. FIO_2_ was set at the lowest level possible while still achieving blood PO_2_ levels within a normal range. To avoid atelectasis, positive end-expiratory pressure (PEEP) was fixed at five cm H_2_O and recruitment was performed regularly by increasing PEEP to 10–15 cm H_2_O. A small venous catheter was placed in one ear vein to facilitate the infusion of fentanyl and propofol as primary anaesthesia during the remainder of the experiment. Throughout the experiment, fluid was administered according to existing guidelines for porcine anaesthesia [19, 20]. A bladder catheter with a thermal sensor was inserted and used for monitoring diuresis and core temperature. To detect any possible arrhythmias, constant electrocardiography was performed. Real-time arterial pressure measurements were achieved using an arterial catheter connected to a pressure transducer inserted into the right carotid artery. The same catheter was used for drawing blood for analysis.

After a full sternotomy, arterial catheters were inserted into the pulmonary artery and the left atrium to measure pressure differences over the pulmonary vascular system. The catheters were also used for drawing blood for analysis. Using an articulating dissection instrument (Wolf Lumitip Dissector™, AtriCure, Mason, Ohio, US)-modified for multiple use as a guide, a division of the fibrous tissue connecting the aorta and the pulmonary artery was made, allowing for the placement of a sonography probe (16–18 mm, MediStim, Copenhagen, Denmark) around the main pulmonary artery. The probe was connected to a flow monitor (MediStim, Copenhagen, Denmark), enabling real-time measurements of the cardiac output.

The flow through the main pulmonary artery was used as cardiac output and for calculating the pulmonary vascular resistance (PVR). PVR was calculated using the following formula: PVR = (80 x (Mean Pulmonary Arterial Pressure- Left Atrial Pressure))/Pulmonary Blood Flow. The right femoral artery and vein were exposed after surgical incision, and after heparin injection (30,000 IE), a 15 French cannula (Medtronic, Minneapolis, Minnesota, US) was inserted into the artery for infusion of blood from the extracorporeal system. Drainage to the system was achieved using a 21 French cannula (Medtronic, Minneapolis, Minnesota, US) inserted over a guide wire into the right jugular vein.

For extracorporeal circulation, we used a prototype centrifugal pump to drive the extracorporeal circulation through an oxygenator (QUADROX adult, Maquet, Rastatt, Germany). Oxygen flow to the oxygenator was set at a constant level of 2 L/min with 100% oxygen. Extracorporeal blood flow was measured using an ultrasonic flowmeter (Sono TT, em-tec GmbH, Finning, Germany). The pump was initially set to 3000 rounds per minute (RPM), resulting in a mean flow of 2.4 L/min. By using two additional Y-connectors, a shunt in the external circulation was created, allowing us to initiate/stop external circulation quickly without tampering with pump settings.

CO was delivered from a pressure cylinder with an attached pressure reduction valve. Through “air” tubes, CO gas was connected to 1) a CO monitor (Exhaust Emission Gas Analyser, Model SV-5Q, China Coal, Shaanxi, China) and 2) the research animal via the ventilator, forming a closed system to avoid leakage of CO into the operating theatre. When administering CO, the valve was opened briefly with intervals to avoid overdosing and to keep the inhalation concentration at a level of approximately 1–2%. CO administration was stopped permanently at the time of randomization. Conventional arterial blood gas analyses were made regularly using a blood gas analyser (ABL800 FLEX Series, Radiometer Medical, Brønshøj, Denmark), allowing us to track changes and to keep track of HbCO during the experiment. Constant CO monitoring with an alarm was used to secure the safety of laboratory personnel in the room.

### Experimental protocol

Animals were randomly assigned to the study groups following simple randomization procedures (computerized random numbers) by a third party. Allocation concealment was kept blinded for the study personnel who were going to implement assignments at the time that cardiac failure was evident (defined as cardiac output decreased to 50%), which was taken as a surrogate measure of severe CO intoxication. At this point, a sequential numbered, sealed, opaque envelope was opened. Blinding to the allocated arm was not possible due to the nature of the experiment. The primary outcome in the model was survival. The histological effect on lung tissue and changes in PVR were used as secondary outcomes.

Cardiac arrest, defined as systolic blood pressure below 25 mmHg, was treated using advanced resuscitation according to the 2015 guidelines of the European Resuscitation Council [21, 22]. However, chest compressions were replaced by internal cardiac compressions, and direct current defibrillation was attempted using internal paddles (Zoll Pro Pac MD, ZOLL Medical Corporation, Chelmsford, Massachusetts, US). If resuscitation failed in the ventilator group (defined as no return of spontaneous circulation (ROSC) and a lack of any signs of improvement in the condition within 10 min), ECMO was established. Weaning from ECMO in both groups was not attempted until HbCO was less than 10%. Weaning was not considered successful unless 10 min of off-pump circulation was completed without cardiac or respiratory failure. All animals were euthanized using pentobarbital intravenous injection after completion of the experiments.

Tissue samples of approximately eight cm^3^ were taken from the lungs close to the pleura at different sites prior to CO poisoning. Similar samples were taken after intoxication at the time of randomization. These samples were preserved using formalin and analysed microscopically at the Department of Forensic Medicine, Aarhus University, after slicing and staining with haematoxylin and eosin (H&E).

### Statistical analysis

We used the “resource equation” method for sample size calculation in the present study, as it was not possible to assume anything about the effect size or to determine standard deviations from previous studies [23]. According to this method, the value “E” was measured by the following formula: E = Total number of animals − Total number of groups. Any sample size that maintained “E” between 10 and 20 was considered adequate. To avoid unnecessary wastage of resources and comply with ethical issues, we kept the number of animals included in this pilot study to six in each group, i.e., “E”: 12–2 = 10.

For statistical analysis, we used the open source freeware program R, version 3.4.3/R-studio and IBM SPSS, version 25. Group comparisons at baseline and at the point of randomization were made using an unpaired t-test. Tests for normality were performed by visual inspection of qq-plots of all variables and Levene’s test for equality of variances. A paired-samples t-test was conducted to compare mean pO_2_ at baseline and mean pO_2_ at the point of randomization. A simple linear regression was constructed to predict pO_2_ based on HbCO. Similarly, we constructed a linear regression to predict pulmonary vascular resistance (PVR) based on HbCO and PVR based on pO_2_. An exponential regression was performed to describe the correlation between lactate and HbCO.

## Results

There were no significant differences between the study groups at baseline (Table 1) and at the time of randomization (Table 2). The mean time of the duration of CO intoxication was 53 min: 51.0 min for the ECMO group (SD = 13.3) and 56.5 min for the ventilator group (SD = 14.8), *p* = 0.97.

**Table 1:** Baseline characteristics

	Ventilator (*n* = 6)		ECMO (n = 6)		Difference	
		CI (95%)		CI (95%)		*P*-value
Weight (kg)	48.7	46.2–51.1	47.5	44.5–50.5	1.17	0.46
HbCO (%)	2.9	2.3–3.5	3.2	2.5–3.8	−2.5	0.47
pH	7.41	7.33–7.49	7.42	7.36–7.47	−0.01	0.86
Hb (mmol/L)	4.58	4.08–5.09	4.87	3.84–5.89	−0.28	0.54
pCO_2_ (kPa)	5.37	4.85–5.88	5.27	4.70–5.84	0.10	0.75
pO_2_ (kPa)	11.53	8.33–14.74	10.68	9.58–11.78	0.85	0.53
Lactate (mmol/L)	1.23	0.72–1.75	1.1	0.68–1.53	0.13	0.62
Temperature (°C)	37.3	35.8–38.9	37	36.6–37.5	0.28	0.67
Cardiac output (L/min)	3.45	2.77–4.13	3.72	2.84–4.60	−0.27	0.55
MAP (mmHg)	78.5	62.9–94.1	85.7	76.1–95.3	−7.2	0.34
HR (beats/min)	71.2	47.5–94.8	73.5	57.2–89.8	−2.3	0.84
MPAP (mmHg)	24.5	23.1–26.0	25.8	20.0–31.6	−1.3	0.58
MLAP (mmHg)	10.2	8.9–11.4	10.5	9.4–11.6	−0.3	0.61
PVR (dyn·s/cm^5^)	344.2	238.7–449.6	325.2	248.8–401.5	19	0.72

The table shows baseline characteristics of essential values in the ventilator group vs the ECMO group. *MAP* Mean Arterial Pressure, *HR* Heart Rate, *MPAP* Mean pulmonary Pressure, *MLAP* Mean left atrial pressure, *PVR* Pulmonary Vascular resistance

**Table 2:** Characteristics at point of randomization

	Ventilator (*n* = 6)		ECMO (n = 6)		Difference	
		CI (95%)		CI (95%)		*P*-value
HbCO (%)	69.7	59.4–80.0	67.9	49.6–86.2	−2.0	0.83
pH	7.26	7.16–7.36	7.31	7.23–7.39	−0.05	0.34
Hb (mmol/L)	5.85	4.98–6.72	6.17	5.21–7.12	−0.32	0.54
pCO_2_ (mmHg)	37.7	29.0–46.4	39.2	34.4–44.0	−1.4	0.72
pO_2_ (mmHg)	34.5	21.5–47.6	24.4	17.4–31.4	10.1	0.12
Lactate (mmol/L)	7.33	6.45–8.22	6.27	5.27–7.26	1.07	0.07
Temperature (°C)	37.4	36.1–38.8	37.1	36.4–37.7	0.35	0.56
Cardiac output (L/min)	1.57	1.17–1.96	1.34	0.43–2.25	0.23	0.57
% of base line	49.5	37.5–61.4	41.33	6.39–76.26	8.13	0.59
MAP (mmHg)	46.3	35.1–57.6	38.8	32.3–45.4	7.5	0.18
HR (beats/min)	102.5	74.8–130.2	108.17	84.4–131.9	−5.67	0.70
MPAP (mmHg)	20,0	16.6–23.5	16.2	12.5–19.8	3.83	0.08
MLAP (mmHg)	8.2	5.6–10.8	7.8	5.4–10.3	0.33	0.82
PVR (dyn·s·cm^−5^)	657.3	316.2–998.5	579.8	207.8–951.9	77.5	0.70

The table shows characteristics of essential values at point of randomization in the ventilator group vs the ECMO group. *MAP* Mean Arterial Pressure, *HR* Heart Rate, *MPAP* Mean pulmonary Pressure, *MLAP* Mean left atrial pressure, *PVR* Pulmonary Vascular resistance

**Fig. 1:**
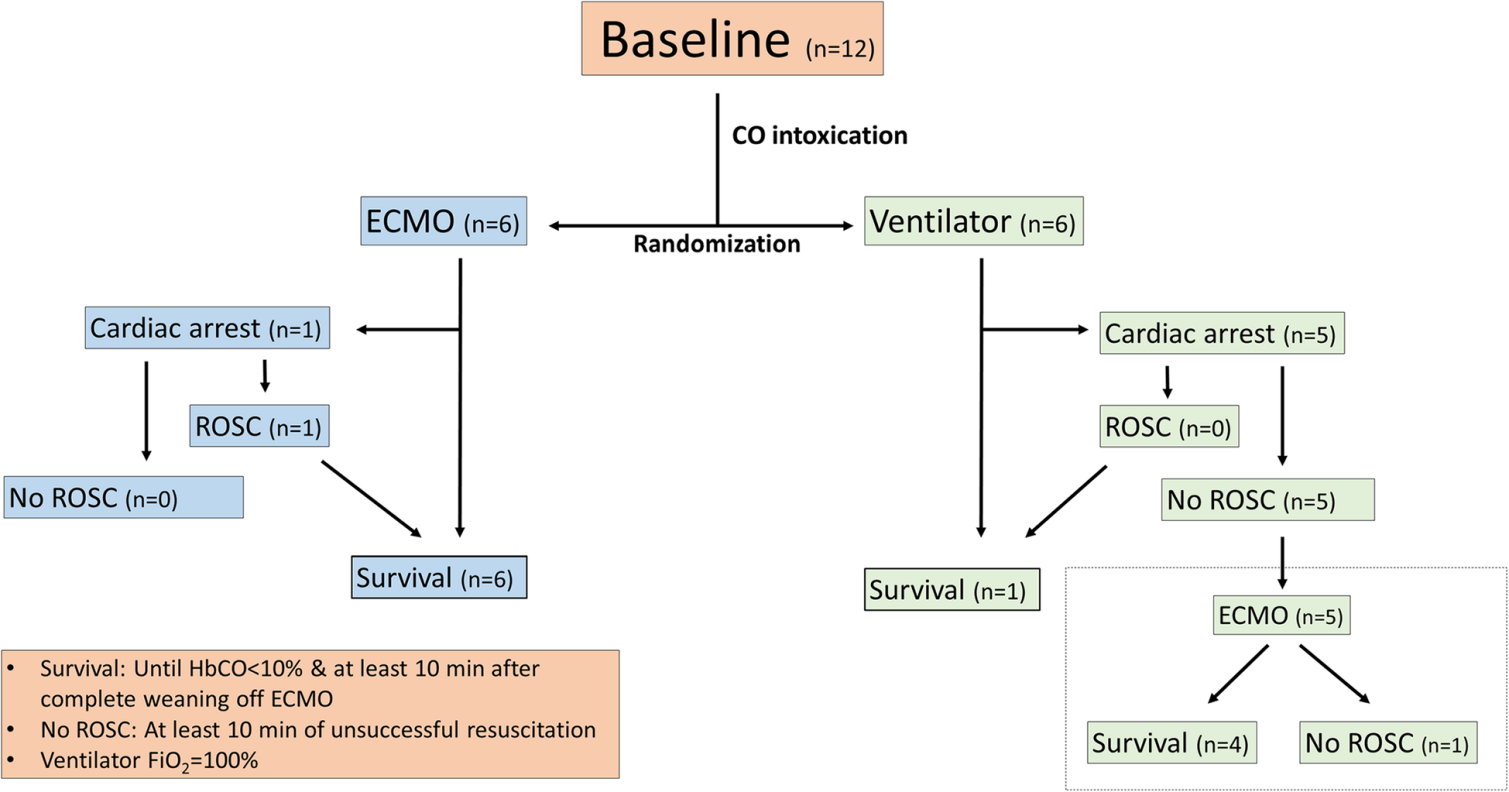
Flowchart of survival. ECMO = Extracorporeal membrane oxygenation, ROSC = Return of spontaneous circulation

All animals survived in the ECMO group for at least 10 min after weaning from ECMO once HbCO was below 10%, although one had to be resuscitated due to a cardiac arrest that occurred immediately after the initiation of external circulation (ROSC after 17 min). The mean time from the identification of heart failure to the initiation of ECMO treatment was 4.3 min. Only one animal survived in the ventilator group, and five suffered from cardiac arrest at an average of 11.8 min after the initiation of treatment. It was not possible to resuscitate any of these animals by conventional means within 10 min of cardiac arrest. However, after initial resuscitation attempts were abandoned, we established ECMO treatment and successfully managed to resuscitate four of these animals (Fig. 1). No adverse events occurred. Time on ECMO was 182.5 min (SD = 21.9) for the ECMO group and 201.6 min (SD = 63.5) for those in the ventilator group that ended up on ECMO after failure of conventional resuscitation, *p* = 0.96. The mean time for HbCO to fall below 0.1 after intoxication was 123.7 min (SD = 20.0) for the ECMO group and 163.7 min (SD = 15.2) for the ventilator group, *p* = 0.56.

**Fig. 2:**
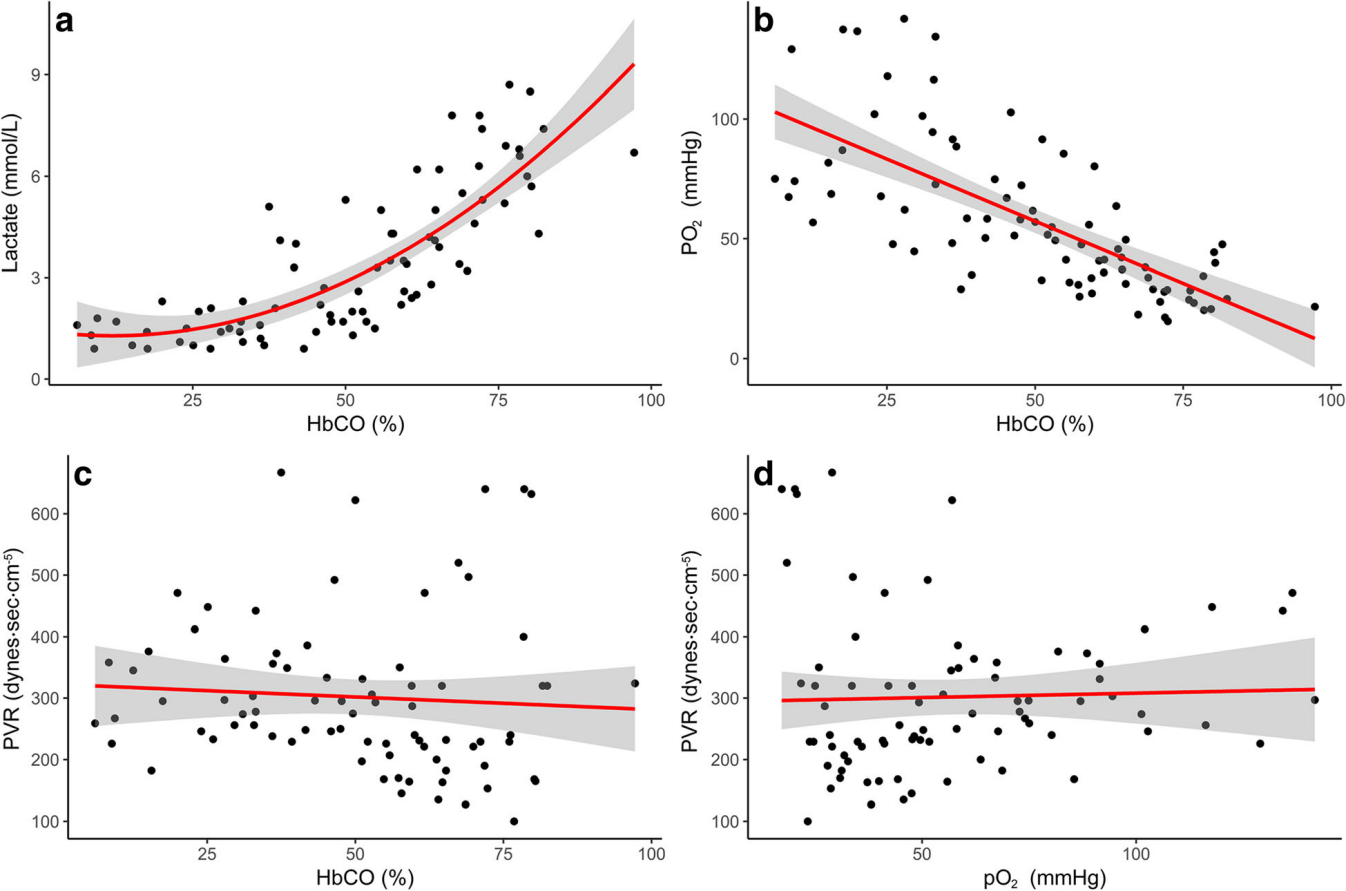
**a** Correlation between blood lactate level and percentage of HbCO. **b** Correlation between the oxygen pressure in the blood and the percentage of HbCO. **c** Correlation between pulmonary vascular resistance (PVR) and the percentage of HbCO in blood. **d** Correlation between oxygen pressure in the blood and PVR

**Fig. 3:**
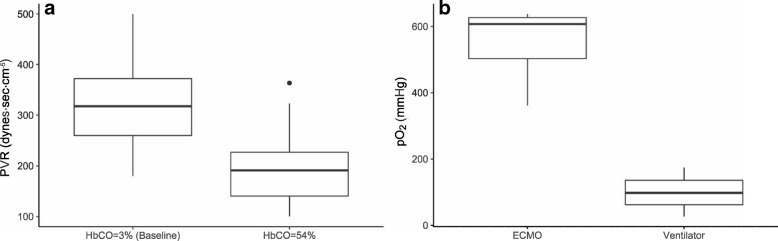
**a** Comparison of pulmonary vascular resistance at baseline (HbCO at 3%) and at the point of heart failure (HbCO at 54%). **b** Highest achieved oxygen pressure dependent on the treatment group

Lactate concentrations in the blood increased exponentially as HbCO increased, *p* < 0.001 (R^2^ of 0.562) (Fig. 2a). A significant regression equation was found when comparing pO_2_ with HbCO, p < 0.001, (R^2^ of 0.496). The predicted pO_2_ was equal to 109.2 mmHg minus 10.4 mmHg for every 0.1% increase in HbCO (Fig. 2b). There was a significant difference in pO_2,_ mean 83.1 mmHg (95% CI: 72.8–93.0) at baseline vs. mean 26.3 mmHg (95% CI: 20.3–26.3) at randomization, *p* < 0.001. We did not find a significant linear regression equation, *p* = 0.54 (R^2^ = 0.008) when exploring the association between PVR and HbCO (Fig. 2c). However, mean PVR was the lowest at HbCO = 54% corresponding to a mean PVR of 200 dyn·s/cm^5^ (95% CI: 148–252) compared to the baseline mean PVR of 319 dyn·s/cm^5^ (95% CI: 261–378) (Fig. 3a). This difference was statistically significant (p < 0.001). No correlation was found between pO_2_ and PVR, *p* = 0.76 (R^2^ = − 0.001) (Fig. 2d). Regarding the highest achieved pO_2_ dependent on treatment, the highest pO_2_ on ECMO was 551.3 mmHg (95% CI: 487.5–615.0) versus the highest pO_2_ on a ventilator at 99.8 mmHg (95% CI: 0–283.5) (Fig. 3b). In surviving animals, we were able to increase pO_2_ from a mean of 27.0 mmHg (95% CI: 19.5–30.8) at the time of randomization to a mean of 209.3 mmHg (95% CI: 102.8–315.0), *p* = 0.003.

**Fig. 4:**
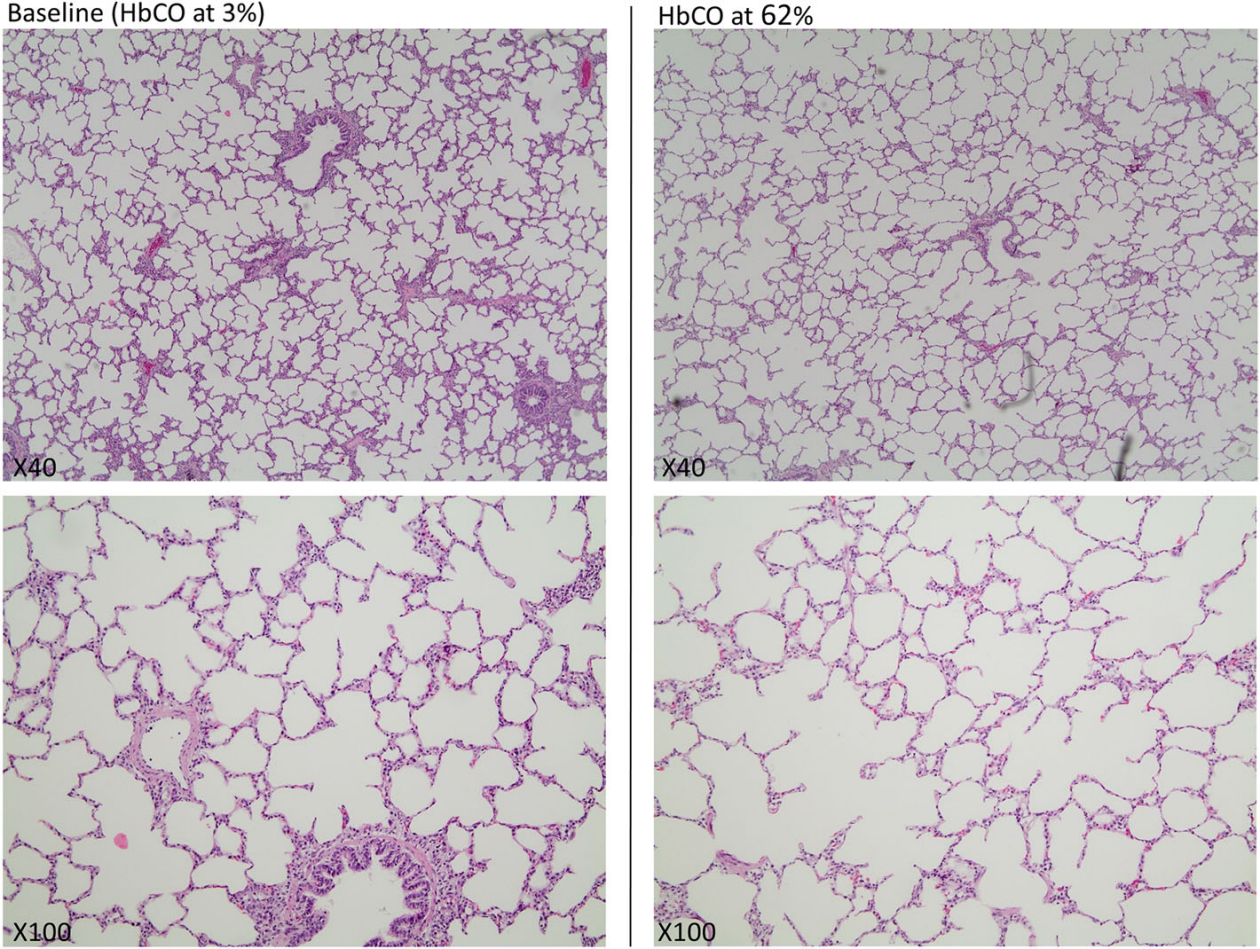
Representative haematoxylin and eosin-stained slices of lung tissue at baseline and at a high level of CO intoxication

There were no microscopic differences in H&E-stained lung tissue biopsies obtained prior to CO intoxication versus after (Fig. 4). No intra-alveolar fluid accumulation and no signs of inflammation were evident.

## Discussion

In this study, we showed that ECMO treatment in severe cases of CO poisoning greatly improved survival compared with conventional resuscitation in an experimental porcine model. Thus, ECMO may serve as a treatment option in addition to conventional treatment following severe CO poisoning. The use of HBO is only possible in a limited number of hospitals within each country, and treatment can only be offered to a fraction of the population without the need for interhospital transportation. In contrast, ECMO treatment is available in mobile systems and can be transferred to the patients [24]. In Denmark, a highly mobile ECMO team exists, using helicopter assets from the Royal Danish Airforce when needed to reduce transport time.

The increased probability of survival, even following cardiac arrest and resuscitation, underlines ECMO treatment’s ability to stabilize respiratory and cardiac function while proper restitution occurs. For practical reasons, we weaned the animals from ECMO as soon as HbCO was below 0.1%. In real clinical settings, more time would probably be advisable to allow for more complete restitution. We chose 10 min post-weaning as marker for survival because in our experience, subsequent circulatory failure would probably reoccur during this timeframe.

A large proportion of CO-poisoned patients may suffer from lung injuries from other components in smoke (e.g., nitrogen oxide gasses, hydrogen chloride) and thermal injuries from the inhalation of hot gases. In these cases, the benefits of ECMO would potentially be even greater, as current treatment with a ventilator and/or HBO rely on the lung diffusion capacity to ensure sufficient oxygen tension in the blood.

In a study of 18 patients who suffered from cardiac arrest due to CO poisoning, none of the patients who were subjected to HBO treatment after resuscitation survived hospitalization [14]. The authors concluded that “the prognosis of this condition should be considered when making triage and treatment decisions for patients poisoned to this severity”, implying that the termination of treatment should be considered if cardiac arrest occurs in this patient category. The cause of this negative outcome may be explained by pulmonary insufficiency due to inhalation injuries from smoke/heat that make efficient gas exchange impossible, and a case report from 2017 indicated that patients with pulmonary insufficiency might experience longer HbCO half-life, diminishing the possible positive benefits of HBO treatment [25]. The results of the present study imply that survival may be possible if ECMO can be established.

The benefits of ECMO may be explained by the release of strain on the heart, lowering oxygen consumption and allowing sufficient restitution following ischaemia. Another favourable effect of using ECMO is its non-dependence on the condition of the lungs and airways. ECMO has the potential to increase blood oxygen tension, diminishing ischaemia and favouring increased formation of oxyhaemoglobin and elimination of CO.

Prior to the experiments, we expected that PVR would increase during CO poisoning, contributing to cardiac insufficiency through a backward failure mechanism. However, this was not the case as the trend was towards a lower PVR during CO poisoning. It is possible that this can be explained by hypoxia induction of the relaxation of smooth muscle cells in resistance vessels in the pulmonary system, but separate experiments must be undertaken to clarify this. To the best of our knowledge, no previous studies regarding changes in PVR due to CO poisoning have been published.

We found a negative linear correlation between HbCO and O_2_. Our initial presumption was that this might be due to a negative impact on the lung tissue, especially the diffusion barrier, making O_2_ absorption progressively harder. This was not supported by the histological findings on the lung biopsies obtained prior to CO poisoning and compared individually with biopsies obtained after CO poisoning; no consistent differences were detected. The answer may be found on a molecular level, undetectable by the analysis of this experiment. Another hypothesis may be that CO causes shunting in the lungs, which is supported by our finding of decreased PVR.

Promising experiments have been made using light to decrease HbCO’s half-life, and it would be simple to expose the oxygenator in the ECMO system to a strong source of light [26]. Other experiments have used O_3_ instead of O_2_ as oxygen supply to the oxygenator in the external circulation to decrease HbCO’s half-life [27]. Some patients suffering from CO poisoning due to inhaling smoke will also suffer from cyanide poisoning [28]. A specific antidote for cyanide may be administered while ECMO stabilizes the patient, the effects of both CO and Cyanide diminish and the patient recovers.

A limitation of this study is that all animals were sacrificed at the end of the experiment due to ethical reasons. Thus, we had no ability to evaluate any neurological outcomes. Additionally, long-term mortality and morbidity could not be evaluated. In two case reports regarding successful ECMO support of patients suffering from severe CO poisoning with insufficient response to traditional ventilator therapy, no neurological deficits were detected during follow-up [29, 30]. There may be a theoretical risk of bias if efforts for resuscitation differed between study groups. However, we have no reason to believe this was the case as we strictly followed published resuscitation algorithms in both groups.

Precautions must be taken when inferring results from animal studies to human clinical settings; nevertheless, since this study involved large animals, we speculate that similar results may be obtained when humans are treated. Furthermore, the benefits of using ECMO must be weighed against the risk of potential complications.

## Conclusion

The use of VA-ECMO following severe cases of CO poisoning with cardiogenic shock greatly improved short-term survival compared with conventional resuscitation in an experimental porcine model. This study forms the basis for further research among patients.

## Abbreviations

CO: Carbon monoxide
ECMO: Extracorporeal membrane oxygenation
FiO_2_: Fraction of Inspired oxygen
H&E staining: Haematoxylin and eosin staining
HBO: Hyperbaric oxygen
NBO: Normobaric oxygen
PEEP: Positive end-expiratory pressure
PVR: Pulmonary vascular resistance
ROSC: Return of spontaneous circulation
RR: Respiratory rate
